# *Pueraria tuberosa* DC Extract Improves Androgenesis and Sexual Behavior via FSH LH Cascade

**DOI:** 10.1155/2013/780659

**Published:** 2013-12-30

**Authors:** Nagendra Singh Chauhan, Vikas Sharma, Mayank Thakur, Alexandra Christine Helena Frankland Sawaya, V. K. Dixit

**Affiliations:** ^1^Department of Pharmaceutical Sciences, Dr. H. S. Gour Vishwavidyalaya, Sagar, Madhya Pradesh 470003, India; ^2^Central Institute for Laboratory Medicine, Charite, Campus Benjamin Franklin, Hindenburgdamm 30, 12200 Berlin, Germany; ^3^Department of Plant Biology, Institute of Biology, State University of Campinas, Campinas, SP 13083-970, Brazil

## Abstract

The aim of this study was to investigate the effects of ethanolic extract of *Pueraria tuberosa* (PT) on sexual behaviour and androgenic activity. Male albino rats were divided into four groups of six animals each: control group 1 (2% acacia solution), PT-treated group 2 (50 mg/Kg), PT-treated group 3 (100 mg/Kg), and PT-treated group 4 (150 mg/Kg). Sexual behavior of male rats in the presence of a female rat was recorded. The treated groups were evaluated for sexual parameters. The extract was characterized using LC-MS. The effect of treatment on anabolic and weight of secondary sexual organs was determined. The histological changes in section of testis and epididymis after treatment were observed. Sperm count in epididymis and fructose content in seminal vesicles were also measured. Levels of hormones like FSH, LH, and T were determined. A dose-dependent increase in sexual behaviors was evidenced in the animals of extract treated groups. Increase in testis weight was recorded in PT. At the highest dose PT also affects the hormones level. The four compounds namely puerarin, daidzein, biochanin-A and formononetin were identified in ethanolic extract using LC-MS. It concluded that PT extract possesses androgenic effect and it significantly increased the sexual behaviour and hormones level.

## 1. Introduction

The importance of sexuality in human life is well recognized in the ancient Indian system of Ayurvedic medicine. An entire category of drugs known as Vajikarana rasayan is devoted to drugs useful in treating sexual deficiencies/disorders [1, 2]. Several plants have been included in the rasayan category which have been explored for their ability to improve sexual function. In recent years, the vajikarana plants, namely, *Curculigo orchioides, Bryonia laciniosa, Asparagus racemosus, Asteracantha longifolia, Chlorophytum borivilianum, Anacyclus pyrethrum, Spilanthes acmella, *and* Dactylorhiza hatagirea,* have been investigated scientifically for their aphrodisiac activity [3–10].

*Pueraria tuberosa* DC (Fabaceae) is a plant widely used in traditional Indian medicine as tonic, aphrodisiac, antirheumatic, diuretic, and galactagogue [11]. *Pueraria tuberosa* is an important constituent of Ayurvedic medicines including Chyawanprash, a popular tonic [12]. *Pueraria tuberosa* is also reported to possess numerous activities like nootropic, antioxidant, and antifertility [13–15]. Interestingly, the plant is considered as a treatment for sexual disorders but on the other hand it is also recognized for its antifertility activity. Since, the doses exhibiting antifertility activity were considerably higher and also because of the absence of any scientific study on the traditionally claimed fertility-enhancing properties of the herb, it was envisaged to carry out a systematic investigation of androgenic and fertility-enhancing properties of the herb along with its possible action on the pituitary and testicular hormone levels.

## 2. Material and Methods

### 2.1. Plant Material

Tubers of *Pueraria tuberosa* were collected from the forest surrounding the campus of Dr. H. S. Gour University, Sagar, Madhya Pradesh, India. The plant was authenticated by Dr. T. R. Sahu at the Department of Botany, Dr. H. S. Gour University, Sagar, India, and a herbarium specimen has been deposited (NSC/H/2008/01).

### 2.2. Preparation of the Extract

Powdered tubers of *Pueraria tuberosa* were first defatted with petroleum ether (60–80°C). The defatted marc was extracted with ethanol (95%) in soxhlet apparatus. Removal of solvent under vacuum yielded ethanolic extracts (3.2% w/w).

### 2.3. Characterization of Extract Using LC-MS

The dried extract was diluted in methanol/water (50 : 50) at a concentration of 1 mg/mL. Sample (5 *μ*L) was injected in a Micromass Waters UPLC-TQD system. A Waters C18 UPLC column with 1.7 *μ*m particles (2 mm × 50 mm) was used. Mass spectrometer conditions were capillary voltage: kV-3.50, cone-30 V, source temperature 120°C, and desolvation temperature 250°C, and nitrogen was used as nebulization gas and argon as collision gas. Collision energy was standardized at 30 for all MS/MS spectra. The flow rate was set to 0.25 mL/min and the linear gradient used was as follows (where A = Milli Q grade purified water with 1% formic acid and B = methanol HPLC grade with 1% formic acid): *t* = 0 min, A : B (99 : 1, v/v); *t* = 0-1 min, A : B (99 : 1, v/v); *t* = 2-3 min, A : B (95 : 5, v/v); *t* = 3–6 min, A : B (80 : 20, v/v)); *t* = 6–9 min, A : B (40 : 60, v/v)); *t* = 9-10 min, A : B (20 : 80, v/v)); *t* = 10-11 min, A : B (5 : 95, v/v)); *t* = 11–14 min, A : B (5 : 95, v/v)); *t* = 14–16 min, A : B (99 : 1, v/v). For quantitative estimation, standard solution of puerarin was used.

### 2.4. Animal

Albino Wistar rats weighing 120–130 g and aged 3-4 months were housed in a standard laboratory condition. They were fed standard pellet diet and kept at 24 ± 2°C and day-night cycle 06:00 h to 18:00 h.

### 2.5. Treatment

The experiment was carried under dim red light and the behavioral aspects were video-recorded for the duration of 60 minutes for each rat using a digital camera (Olympus, EX120). Observational and behavioral analysis was performed in a wooden chamber with a glass wall (70 × 40 × 60 cm) under diffused red light in the dark phase of the light-dark cycle. The chamber had a special small opening at the side for introducing the female as stimulus. The video-recorded data was subjected to analysis using freeware version of EthoLog v 2.2.5 E.B. Ottoni, (Sao Paulo Brazil) run on Windows Xp. The rats were divided into four groups of 6 male rats. Group I animals served as control and received only vehicle, that is, 0.2% gum acacia suspension. Animals of group II, III, and IV were given ethanolic extract orally by feeding needle 50 mg/kg (PT50), 100 mg/kg (PT100), and 150 (PT150) mg/kg, respectively, for 28 days.

### 2.6. Effect on Male Sexual Behaviour

The effect on sexual behavior of male rats was evaluated after 28 days of treatment with various doses of PT to respective groups. In brief, a male rat was placed in the observation glass chambers to acclimatize with the cage environment. After about 10 min, a sexually receptive female rat was dropped silently from one side of the chamber as a stimulus. The sexual behavior, for example, mount frequency, intromission latency, postejaculatory latency, and mount latency, was recorded. Mount latency (ML) was calculated as the time between the introduction of female and the occurrence of first mount; mount frequency (MF) was observed as total number of mounts during the period of observation; intromission latency was considered as the time for first intromission after introduction of female in the cage; postejaculation latency was calculated as the lag time between first intromission and the next within 30 min [2].

### 2.7. Effect on Sexual Organ Weight and Histological Studies

After 28 days of treatment as described above, the body weights of animals were taken after which animals were killed by decapitation. Testis, seminal vesicles, epididymis, and prostate glands were carefully removed and weighed. Testis and epididymis of animals were cut into small pieces and fixed in bovine's fixative. After dehydration with varying percentage of ethanol, sections were cut (6 *μ*), stained with haemotoxylin and eosin, and then analyzed microscopically [4].

### 2.8. *In Vivo* Sperm Count

Epididymes of rats of each group were homogenized and taken into 5 mL of 1% sodium citrate solution and crushed thoroughly with the help of needle and forceps until a milky suspension was obtained. The solution was filtered through 80 *μ* mesh and the volume was made up to 10 mL with the same solution; the made-up volume was inclusive of washing of the filter. The suspension was shaken thoroughly and the spermatozoa were counted in counting chamber of the haemocytometer. The count was cross-checked by a person blinded to treatment group [4]. The average numbers of sperms per chamber are reported using the following formula: total number of sperm = *X* × 10^6^ where *X* is the total number of sperm counted in all Square.

### 2.9. Fructose Content in Seminal Vesicles

The seminal vesicles were macerated with 3 mL of distilled water and centrifuged at 4000 rpm for 12 min. To the supernatant fluid collected after centrifugation, 0.5 mL of resorcinol and then 1.5 mL of HCl was added. The mixture was kept at 80°C for 12 min. The reaction with resorcinol developed a rosy color which was measured at 500 nm using spectrophotometer. A calibration curve was drawn using dilutions of fructose solution and measurement of the color developed with resorcinol and HCl [4].

### 2.10. Hormone Assay

After 28 days of treatment, the animals were killed by decapitation; the blood was extracted and serum was separated by centrifugation at 1000 g for 5 minutes. Isolated serum was further used for hormone level determination. Serum concentration of testosterone (EIAgen Testosterone kit, Italy, Import Lic no. NCD-164D), luteinizing, and follicle-stimulating hormones (ERBA Fertikit, Germany, Import Lic no. NCD 175/2006; lot numbers are 80425.7and 71210.10) was measured by following an immunoenzymatic method with an ELISA reader, according to the commercial protocol as defined in the kit, without any further modification.

### 2.11. Statistical Analysis

Results are reported as mean ± SD. The treated groups were compared to control by ANOVA following Dunnet's test. Significance level was set at *P* < 0.05 and confidence level at 95%. Statistical analysis was carried out using Instat v 2.1 software residing in a Pentium IV processor run on Windows Xp.

## 3. Results

### 3.1. LC-MS Profile of Extract

**Figure 1:**
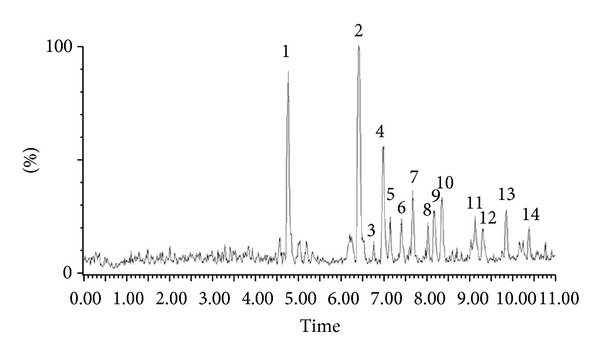
LC Chromatogram of ethanolic extract of *Pueraria tuberosa. *

**Figure 2:**
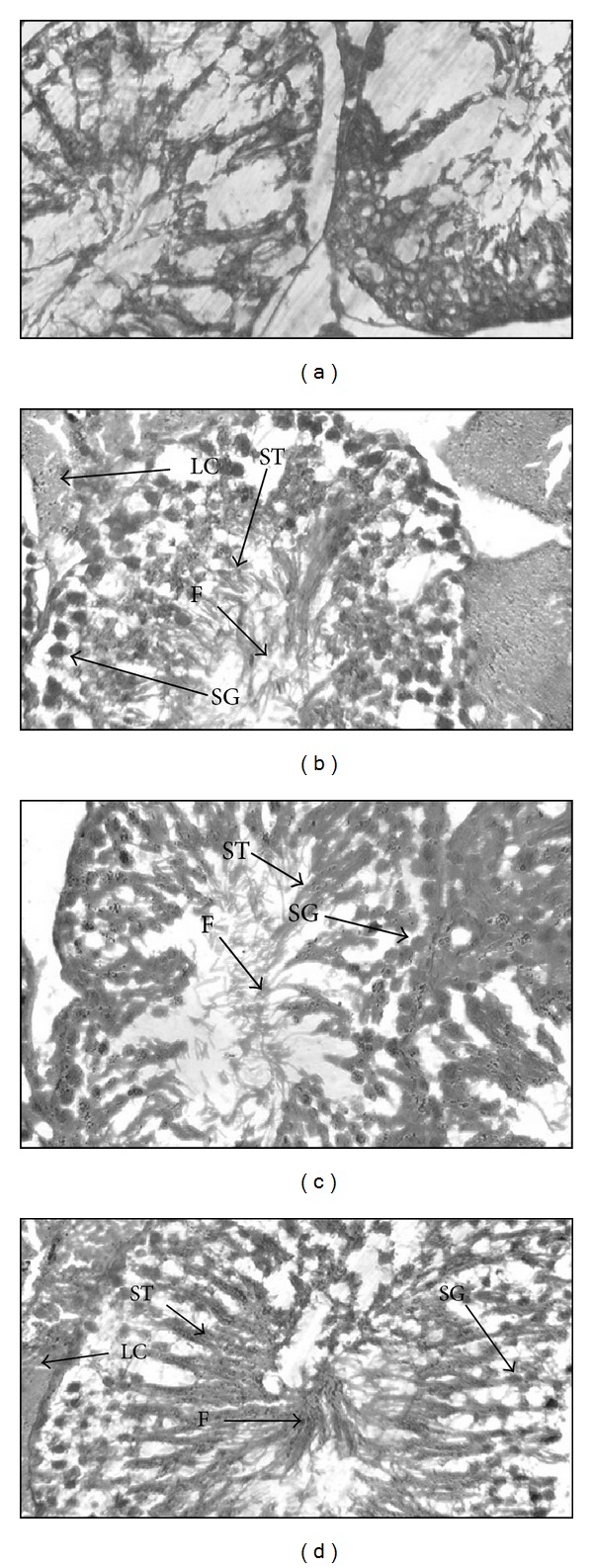
Effect of ethanolic extract of *Pueraria tuberosa* on histology oftestis. (a) Control group. (b) Ethanolic extract 50 mg/kg. (c) Ethanolic extract 100 mg/kg. (d) Ethanolic extract 150 mg/kg treated groups. F; fagellae; LC; Leydig cells; SG; spermatogonia; ST; spermatid.

The extract of *Pueraria tuberosa* was injected in the UPLC-TQD system under a binary gradient solvent system. The representative HPLC/MS chromatograms for ethanolic extract of *Pueraria tuberosa* are shown in Figure 1. A total of fourteen compounds were detected by HPLC and four of them were characterized by MS (Figures 2(a) and 2(b)). Based on mass spectral and compared with the literature data [16–21], the compounds which are identified are in Table 1.

From the MS^n^ spectra of peak 1, the fragmentation of the deprotonated ion of m/z 415 resulted in product ions of m/z of 295 and 267 which were ascribed to the loss of C_4_H_8_O_4_ and C_4_H_8_O_4_ –CO–H. Therefore, peak 1 was identified as puerarin by comparison with the literature and a standard used in the study.

For peak 2, the fragmentation of the deprotonated ion of m/z 253 and gave product ions of m/z 224, 223 [M–CH_2_O–H]^−^, 208, and 133. Therefore, peak 2 was identified as daidzein by comparison with the literature data.

For peak 3, the fragmentation of the deprotonated ion of m/z 283 gave product ions peaks of m/z 267, 253, and 240. The fragmentation pattern was similar to biochanin-A as reported in the literature.

For peak 5, the fragmentation of the deprotonated ion of m/z 267 and gave product ions of 252, 239, 223, and 135. The fragmentation pattern suggests that the compound could be formononetin.

Biochanin-A and formononetin were first time identified in the tubers of *Pueraria tuberosa*. The percentages of compounds calculated on the basis of peak area were puerarin (0.042), daidzein (0.063), biochanin-A (0.003), and formononetin (0.008), respectively, in the ethanolic extract of *Pueraria tuberosa*.

### 3.2. Sexual Organ Weight and Histological Studies

PT treatment resulted in an increase in the body weight of treated animals. Significant increase in weights of sexual organs like testes, prostate, seminal vesicles, and epididymis was observed in case of PT 100 and PT150, while in case of PT50 there was a nonsignificant increase. This effect was thus dose dependent in the studied doses and is described in Table 2.

### 3.3. Sexual Performance

PT treatment had a marked influence on sexual behavior of animals and in case of all the parameters evaluated there was a clear indication of dose dependence in improving the sexual behavior in rats. The mount latency and postejaculation latency was significantly reduced, while a three-fold increase in mount frequency was recorded with 150 mg/kg dose of PT. The results for effect of PT 50, 100, and 150 treatments are described in Table 3.

### 3.4. Testis

Transverse sections (TS) of testes of control group animals showed normal histoarchitecture. The Sertoli and Leydig cells of normal size were present. The seminiferous tubules were normal in number with bundles of spermatozoa. In the TS of PT-treated animals an increase in diameter of seminiferous tubules was observed. Extract treatment also resulted in an improved spermatogenesis in all groups as compared to control group (Figure 2).

### 3.5. Epididymis

**Figure 3:**
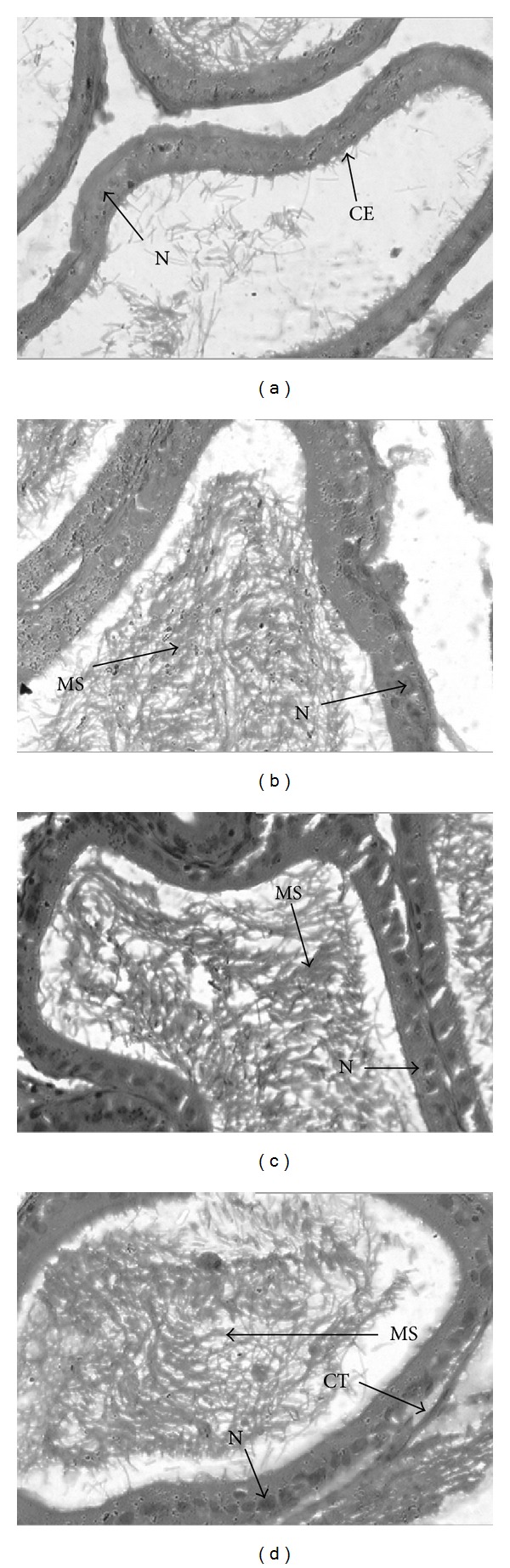
Effect of ethanolic extract of *Pueraria tuberosa* on histology of epididymis, (a) Control group. (b) Ethanolic extract 50 mg/kg. (c) Ethanolic extract 100 mg/kg. (d) Ethanolic extract 150 mg/kg treated groups. MS: mobile spermatozoa; N: nucleus; CE: cuboidal epithelium; CT: connective tissue.

In the epididymides of control rats, the short columnar epithelium consisted of principal cells with elongated nucleus. Basal (cuboidal) and principal (columnar) cells of the epithelium showed a pseudostratified appearance with apical stereocilia. The nucleus of the former cell type was situated basally and that of the latter cell type more centrally. The lumen of epididymis also shows scattered spermatozoa (Figure 3). In comparison, the epididymis of PT-treated animals showed an increase in the size of the tubules. The lumen contained relatively large packed spermatozoa. The effect was most marked in case of PT150-treated animals.

### 3.6. Fructose Content

The treatment with ethanolic extract influenced the fructose concentration in the seminal vesicles and a marked increase in fructose concentration was observed in treated animals. A 24.17% increase in fructose concentration was recorded with PT 150 treatment in comparison to control group animals (Table 4).

### 3.7. Sperm Count

The *in vivo* sperm count in control and extract test groups is presented in Table 4. There was a significant increase in the number of sperms in extract treated groups as compared to control.

### 3.8. Hormone Level

**Figure 4:**
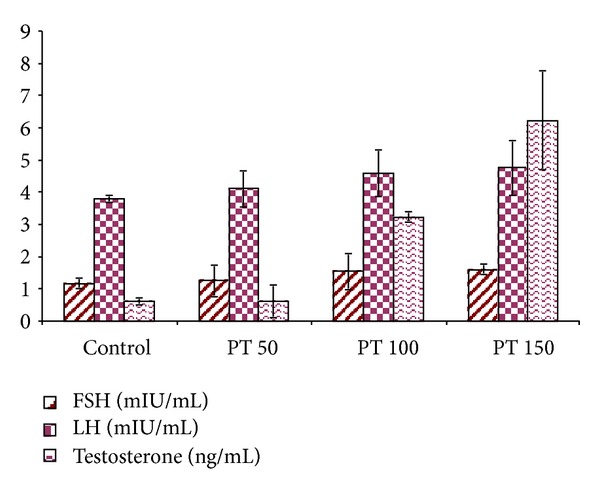
Effect of ethanolic extract of *Pueraria tuberosa* on hormones level.

Elevated hormone levels were recorded in animals treated with PT. The serum testosterone, luteinizing hormone, and follicular-stimulating hormone-levels were significantly increased in all the treated groups. There was a dose-dependent effect of the treatment with the following order: PT50 < PT100 < PT150 (Figure 4).

## 4. Discussion

This is the first report on effect of ethanolic extract of *P. tuberosa* on sexual behaviour of male rats and its effect on hypothalamic pituitary gonadal axis. The extract treatment for 28 days to rats significantly improved the androgenic and sexual behaviour parameters. There was also an increase in serum concentration of FSH and improvement in serum testosterone level in group treated with PT. Administration of PT showed a significant androgenic stimulation as evidenced by an increase in the weights of the testis, epididymis, and seminal vesicles. Spermatogenesis was also improved and is evidenced by improvement in the histoarchitecture of testicular sections. It can therefore be stated that the weight gain in secondary sexual organs is correlated with increased levels of serum FSH and testosterone by PT.

Recently, reports on extracts of *Lycium barbarum* fruit extract (10 mg/kg), *Massularia acuminata* stem extract (250, 500 and 1000 mg/kg), *Satureja khuzestanica* essential oil (75, 150, and 225 mg/kg/day) [22–24], and *Bryonia laciniosa* seed extract (50, 100, and 150 mg/kg) [4] administered to rats have shown an increase in serum hormone level (FSH, LH, T) and accessory sexual organ weight. In one of the allied species of PT known as *Pueraria mirifica* containing phytoestrogens similar to PT, there was an increase in the level of LH and FSH along with an improved mating efficiency [25]. Phytoestrogen puerarin has been reported in the roots of *Pueraria tuberosa* [26, 27].

A number of scientific investigations have shown that phytoestrogen compounds exert biological activity via the central nervous system [28]. Amongst other pharmacological properties of phytoestrogens are their antioxidant, neuroprotective, antidepressant, and anxiolytic activities. Phytoestrogens present in PT might be contributing to the improvement of sexual behavior in rats. Phytoestrogens like daidzein and genistein also affect neurobehavioural actions are largely antioestrogenic, either antagonising or producing an action in opposition to that of oestradiol [29]. The extract might be acting through is the stimulation of endogenous estrogens synthesis which is a contributing factor in male fertility [30]. Increases in LH, FSH, and testosterone levels also indicate an effect of PT on gonadotropin release hormone GnRH. GnRH agonist effect may be the mechanism involved in the androgenic and estrogenic activities evidenced in male rats. It is via the same mechanism that the antifertility activity in female rats can be explained via the overproduction of estrogen where a feedback mechanism for overproduction of GnRH may be a guiding principle. The present study provides evidence for the role of phytoestrogenic compounds from PT in improvement of sexual function and testosterone production in male rats and thus adds to the evidence for its ethnopharmacological utilization as an Ayurvedic herb for improvement of sexual performance and fertility.

## Figures and Tables

**Table 1:** The structure of compounds identified in *Pueraria tuberosa* by LC-MS.

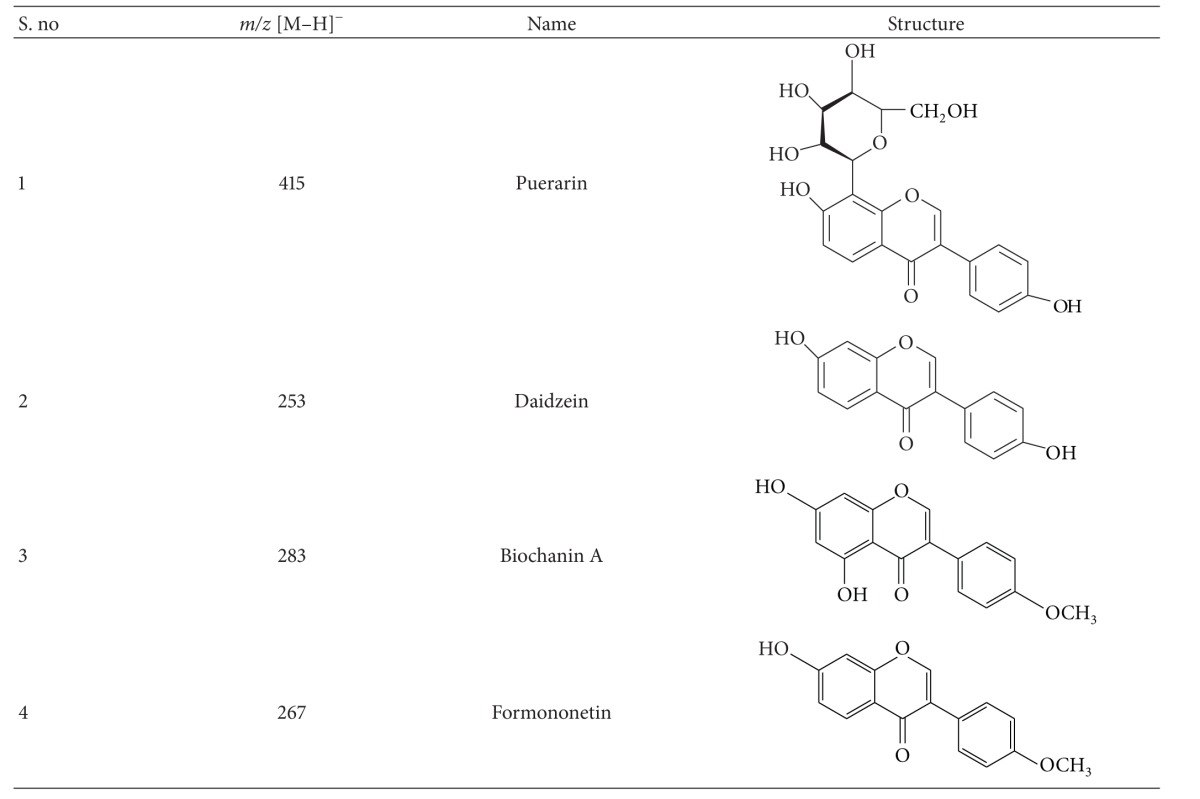

**Table 2:** Effect of ethanolic extract of *Pueraria  tuberosa* on body and sexual organ weights.

Group	Body weight of animals on first days (gm)	Body weight of animals on 28th day (gm)	Testis weight relative to body weight (mg/100 g)	Prostate weight relative to body weight (mg/100 g)	Seminal vesicles weight relative to body weight (mg/100 g)	Epididymis weight relative to body weight (mg/100 g)
Control	128.43 ± 4.25	133.83 ± 3.25	955.5 ± 5.4	102.6 ± 2.4	623.1 ± 2.1	455.4 ± 3.8
PT 50	127.27 ± 1.22	138.12 ± 2.24	1052.1 ± 4.2	105.2 ± 1.4	626.1 ± 1.14	460.1 ± 2.4
PT 100	127.21 ± 3.26	141.24 ± 1.46**	1134.2 ± 2.8	108.4 ± 1.4	632.1 ± 2.4**	468.1 ± 1.2**
PT 150	126.92 ± 2.12	151.36 ± 3.24**	1232.1 ± 3.2**	111.4 ± 3.2**	636.2 ± 1.2**	476.2 ± 2.4**

*F* value		7.894	855.41	2.957	10.752	12.224

Results are expressed as means ± SE.

**P < 0.01 compared with control.

One-way ANOVA followed by Dunnett's test comparing all versus control.

**Table 3:** Effect of ethanolic extract of plants on sexual behavior.

Groups	Mount frequency	Mount latency (seconds)	Intromission latency (seconds)	Postejaculatory latency (seconds)
Control	4.16 ± 1.32	267.33 ± 36.25	359 ± 29.30	569.33 ± 13.06
PT 50	8.00 ± 0.25	204.66 ± 2.61**	345.83 ± 2.81	550.33 ± 3.24
PT 100	11.85 ± 0.30**	173.33 ± 2.81**	319.50 ± 2.82	509.66 ± 2.70**
PT 150	13.0 ± 0.51**	152.33 ± 2.49**	307.83 ± 3.06	492.00 ± 3.14**

*F* Value	29.98	7.928	2.499	25.679

Results are expressed as means ± SE.

**P < 0.01 compared with control.

One-way ANOVA followed by Dunnett's test comparing all versus control.

**Table 4:** Effect of ethanolic extract of plants on the concentration of fructose in seminal vesicle and sperm count.

S. no.	Treatment groups	Fructose contents (mg/g)	Sperms count (millions/mL)
1	Control	2.11 ± 0.24	120.2 ± 1.8
2	PT 50	2.40 ± 0.28	124.6 ± 1.2
3	PT 100	2.44 ± 0.14	130.2 ± 2.4**
4	PT 150	2.62 ± 0.24	132.1 ± 1.2**

	*F* value	0.8372	9.882

Results are expressed as means ± SE.

**P < 0.01 compared with control.

One-way ANOVA followed by Dunnett's test comparing all versus control.
